# Evaluation and modeling of direct membrane-feeding assay with *Plasmodium vivax* to support development of transmission blocking vaccines

**DOI:** 10.1038/s41598-020-69513-x

**Published:** 2020-07-28

**Authors:** Kazutoyo Miura, Bruce J. Swihart, Michael P. Fay, Chalermpon Kumpitak, Kirakorn Kiattibutr, Jetsumon Sattabongkot, Carole A. Long

**Affiliations:** 1 Laboratory of Malaria and Vector Research, National Institute of Allergy and Infectious Diseases, National Institutes of Health, 12735 Twinbrook Parkway, Rockville, MD 20852 USA; 2 Biostatistics Research Branch, National Institute of Allergy and Infectious Diseases, National Institutes of Health, Rockville, MD USA; 3 Mahidol Vivax Research Unit, Faculty of Tropical Medicine, Mahidol University, Bangkok, Thailand

**Keywords:** Parasitology, Vaccines

## Abstract

Standard and direct membrane-feeding assays (SMFA and DMFA) are fundamental assays to evaluate efficacy of transmission-blocking intervention (TBI) candidates against *Plasmodium falciparum* and *vivax*. To compare different candidates precisely, it is crucial to understand the error range of measured activity, usually expressed as percent inhibition in either oocyst intensity (% transmission reducing activity, %TRA), or in prevalence of infected mosquitoes (% transmission blocking activity, %TBA). To this end, mathematical models have been proposed for *P. falciparum* SMFA (PfSMFA), but such study for DMFA is limited. In this study, we analyzed *P. vivax* DMFA (PvDMFA) data from 22,236 mosquitoes tested from 96 independent assays. While the two assays are quite different, a zero-inflated negative binomial (ZINB) model could reasonably explain the PvDMFA results, as it has for PfSMFA. Our simulation studies based on the ZINB model revealed it is better to report %TRA values with a proper error range, rather than observed %TBA both in SMFA and DMFA. Furthermore, the simulations help in designing a better assay and aid in estimating an error range of a %TRA value when the uncertainty is not reported. This study strongly supports future TBI development by providing a rational method to compare different candidates.

## Introduction

According to the World Malaria Report 2019, 228 million clinical cases and 405,000 deaths were estimated due to malaria in 2018^1^. Of the five *Plasmodium* species which infect humans, *P. falciparum* is the most abundant and deadly species, especially for < 5-year old children who live in sub-Saharan Africa regions. *P. vivax* malaria is the second major contributor to global clinical malaria, and it is geographically widespread. *P. vivax* had been considered as a "benign" malaria for a long time, but recent studies have shown that it can cause severe consequences including death^2^. While existing anti-malarial control measures, such as insecticide-treated nets, rapid diagnosis, and antimalarial drugs, have reduced malaria cases and deaths dramatically in the last two decades, the numbers are similar between 2014 and 2018^1^. Therefore, in addition to expanding applications of current control measures, efforts must to be made to develop new tools, such as transmission-blocking vaccines (TBVs)^3^.

For the development of TBVs as well as transmission-blocking drugs (TBD), an assay which can evaluate reduction or complete blocking of parasite growth in mosquitoes is indispensable. The standard membrane-feeding assay (SMFA) or direct membrane-feeding assay (DMFA) have been used widely for the development of transmission-blocking interventions against both *P. falciparum* and *P. vivax*^3–9^. The membrane-feeding assays have been also utilized to evaluate transmission-reducing immunity induced by natural malaria infections^10,11^ and to determine vector competence in transgenic mosquitoes^12^, which is one of the alternative strategies to reduce malaria transmission in the field. SMFA is conducted with cultured sexual-stage parasites (gametocytes), while DMFA uses blood samples from *P. falciparum*- or *P. vivax*-infected patients as the source of gametocytes. Since there is no reliable methodology to transport gametocytes for a long distance without losing their infectivity, usually DMFA is performed at (or near) the field site where the blood samples are collected. It is more challenging to control the quality of assays in the field site compared to an assay performed at fully equipped laboratories with updated infrastructure. In addition, different blood samples have different density and/or maturity of gametocytes, and different male-and-female gametocyte ratios, all of which affect infectivity to mosquitoes^13^. SMFA can be performed in more controlled conditions, but on the opposite side, it is difficult to mimic the complexity of parasites seen in the field. For *P. falciparum*, the great majority of SMFAs have been done with NF54 (or the clones from NF54, e.g., 3D7) strains of parasites, and only a limited number of other strains are applicable for SMFA, because strains which can consistently generate high enough gametocytemia in in vitro cultures are rare. For *P. vivax*, it is impossible to prepare cultured gametocytes, so DMFA is the only option at this moment.

At the early stage of TBV and TBD development, it is almost routine to compare biological activity of different candidates (e.g., different antigens, adjuvants, drugs); thus determination of the error range for the measured activity, usually expressed as % inhibition in oocyst density (also called "transmission-reducing activity", or "%TRA") or % inhibition in prevalence of infected mosquitoes (also referred to as "transmission-blocking activity", or "%TBA"), is essential. However, in many studies, observed % inhibition values were reported without proper error ranges (e.g., 95% confidence interval, 95% CI) and a non-parametric statistical test (e.g., a Mann–Whitney test) was used in many cases to compare different groups.

While the approach is reasonable to compare the candidates tested within the same assay, it is almost impossible to properly compare different candidates tested in different feeding assays (even within a single paper) when the assays show different levels of mean oocyst in the control groups.

Early studies in the 1990’s have shown that distribution of oocysts for rodent (*P. berghei*), chicken (*P. gallinaceum*), and human (*P. falciparum* and *P. vivax*) malarias in various *Anopheles* mosquitoes can be better explained by a negative binomial (NB) model than a normal (Poisson) distribution^14,15^; recent studies have demonstrated that a zero-inflated negative binomial (ZINB) model is better than a NB model for SMFA with *P. falciparum* (PfSMFA)^16,17^ and *P. berghei*^16^. In addition to the ZINB models, a beta-binomial model has been used in another study for PfSMFA^18^. These mathematical models allow the calculation of uncertainty around the measured %TRA (e.g., 95% CI) and support a rational comparison of multiple candidates in SMFA. A recent study for DMFA with *P. vivax* (PvDMFA) again demonstrated that the oocyst data deviated from a Poissonian prediction^19^. However, no mathematical model has been published to interpret DMFA data. Our basic hypothesis was that a ZINB model, which has been shown to be useful for PfSMFA, could be universally utilized to analyze the results of any membrane-feeding assay. If this hypothesis is true, the ZINB model can support better designing, reporting and interpreting of all membrane-feeding assays. To this end, in this study, we compared two distinct membrane-feeding assays with human malaria parasites, SMFA with *P. falciparum* NF54 strain using *Anopheles stephensi* mosquitoes (the details of model fitting have been published previously^17,20^) and DMFA with *P. vivax* using *An. dirus* mosquitoes (new data collected from 22,236 mosquitoes in 96 independent assays). While there were significant differences in the best-fit parameters between the two assays, PvDMFA data could be explained reasonably well by a ZINB model, as is PfSMFA. A brief explanation of each parameter of the ZINB model and abbreviations used in this manuscript are summarized in Table 1. We then evaluated the impact of each parameter on the accuracy of % inhibition estimates. Lastly, we simulated how modifications to the assay design (e.g., number of mosquitoes examined per group, performing repeat PvDMFA for the same samples) change the error range of %inhibition estimates. The simulation results will not only support designing new DMFA and SMFA experiments, but also help rational comparisons for transmission-reducing activities among different candidates when the proper error of measurement is not reported.

**Table 1:** Explanation of each parameter in the ZINB model and terminologies used in this manuscript.

Terminology and abbreviation	Description
m_o_-contl	Mean (arithmetic mean) oocyst in control. Each membrane-feeding assay has a unique m_o_-contl value. Table 2 shows the best estimate (and the 95% confidence interval) of m_o_-contl from all DMFA or SMFA data which match the indicated criteria when control antibodies were tested
π	Zero-inflation parameter. It indicates the proportion of mosquitoes without any oocyst in each COM when an inactive sample (i.e., true inhibitory activity is zero) is tested. Higher the number means more uninfected mosquitoes are expected in test and control COMs, regardless of biological activity of a test sample
θ	Negative binomial dispersion parameter. It indicates how the relationship between mean and standard deviation (SD) in oocyst intensity diverges from a Poisson distribution.θ = 1 denotes a Poisson distribution, and values larger than 1 indicate larger SD than expected from the Poisson model
Vf	Variance of the random effects for feed, also called feed-to-feed variation. It indicates how much variation in oocyst intensity is expected when the same sample is tested in different feeding experiments. Vf = 0 denotes no feed-to-feed variation, and larger values mean more variation
Vc	Variance of the random effects for COM, also called COM-to-COM variation. It indicates how much variation in oocyst intensity is expected when the same sample is tested in different COMs in a single assay. Vc = 0 denotes no COM-to-COM variation, and larger values mean more variation
COM	"Container of Mosquitoes”. A group of mosquitoes which are housed in the same container (e.g., a pint cup) and are fed the same blood-test (or control) antibody mixture
95%CI length	The difference between high and low end of 95% confidence interval (CI). For example, when 95% CI of inhibition of a test sample is estimated as 60 to 85%, the 95% CI length of the test is 85–60 = 25. Bigger the 95% CI length means more uncertainty around the estimated inhibition level
TRA	"Transmission-Reducing Activity" or inhibition in oocyst density. In this paper, the inhibitory activity is expressed as percent of TRA, "%TRA"
TBA	"Transmission-Blocking Activity" or inhibition in prevalence of infected mosquitoes. In this paper, the inhibitory activity is expressed as percent of TBA, "%TBA"

## Results and discussion

### Establishment of PvDMFA-specific model

*Plasmodium falciparum* SMFA (PfSMFA) results have been shown to be explained well with a zero-inflated negative binomial (ZINB) model^16,17^. To assess whether a similar ZINB model is applicable for *P. vivax* DMFA (PvDMFA), the correlations between mean and standard deviation (SD) of oocyst intensity, and between mean oocyst intensity and prevalence of infected mosquitoes were evaluated first. For the analysis, PvDMFA data from 96 independent assays with 22,236 mosquitoes tested in a total of 1,022 "Container of Mosquitoes" (COM) were utilized. COM means a group of mosquitoes which were housed in the same container and were fed the same blood-test (or control) antibody mixture. The average number of mosquitoes per COM was 21.8. While more PvDMFA data were available, we only used data with at least one infected mosquito in each COM (if there is no infected mosquito, there is no difference between PvDMFA and PfSMFA, as mean, SD and prevalence are all zero). The original PvDMFA data are seen in Supplementary Table S1.

**Figure 1:**
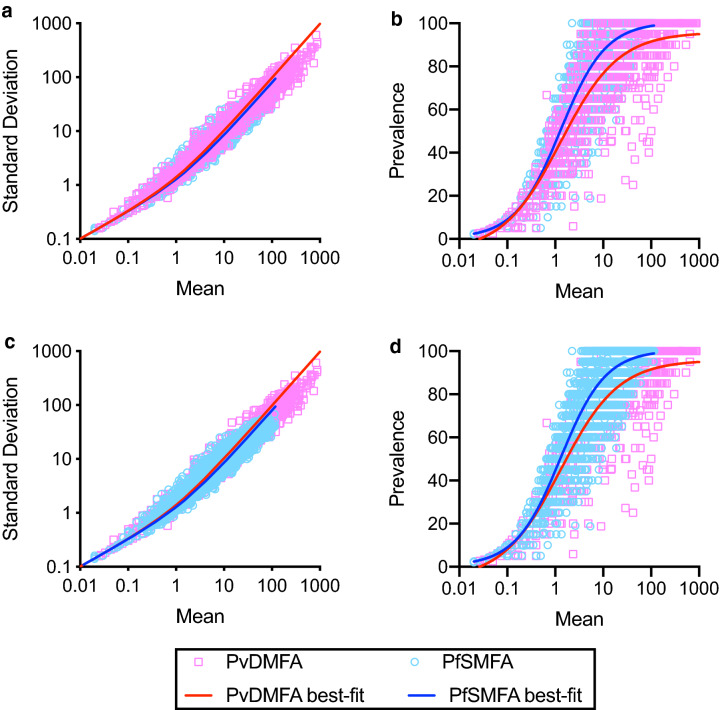
Strong correlations between mean and standard deviation, and between mean and prevalence both in PvDMFA and PfSFMA. Mean oocyst intensity, standard deviation, and prevalence of infected mosquitoes were calculated in each COM from PvDMFA and PfSMFA. Each dot represents data from a single COM (1,022 COMs for PvDMFA and 3,263 COMs for PfSMFA). COM refers to “Container of Mosquitoes”, which were housed in the same container and were fed the same blood-test (or control) antibody mixture. (**a**,**c**) show the same data, but PvDMFA data are presented on top of PfSMFA data in (**a**) and reversed in (**c**). (**b**,**d**) display in the same way. The best-fit curves are shown with individual COM data.

The PvDMFA were performed with *P. vivax* gametocytes collected from *P. vivax* patients and laboratory-reared *An. dirus* mosquitoes, while the PfSMFA were all performed with cultured *P. falciparum* (NF54) gametocytes and laboratory-reared *An. stephensi* mosquitoes. Although there were obvious differences in methodologies, as shown in Fig. 1a,c, the best-fit curves (calculated by a negative binomial fit) for mean-SD associations in two different assays overlapped almost perfectly. While the negative binomial fit line for the PvDMFA data appears to be too high at higher mean oocysts (> 100 oocysts), still the model fits the data reasonably well (R^2^ = 0.951). For mean-prevalence correlations (Fig. 1b,d), the best-fit curves (by a sigmoid curve fit) were similar overall, but the PvDMFA curve shifted slightly to the right. Strong, and similar associations of mean-SD and mean-prevalence were reported in SMFA with rodent malaria^14,16^ and DMFA with *P. falciparum* parasites^21,22^ tested in multiple anopheline mosquitoes. The previous and current studies indicate that ZINB models could be usefully used to analyze feeding assays performed with multiple strains of parasites and mosquitoes, while each parameter value needs to be optimized depending on assays. The similarity (mean-SD and mean-prevalence associations) in different assays suggest that a beta binomial (BB) model could also be used for PvDMFA data as shown in a recent paper with PfSMFA^18^. Both the beta-binomial and negative-binomial models are ways to handle overdispersed count data. The beta-binomial model is for proportions and it requires a maximum count to be declared, while the negative binomial model addresses counts and does not require the maximum count declaration. The "zero-inflated" negative binomial has an additional parameter that allows estimating the proportion of mosquitoes that would never develop oocysts under any circumstance. It is mathematically easy to compare ZINB and NB models, because the NB model is nested within the ZINB model. However, since BB and ZINB models are completely different ones, it is difficult to examine which model is significantly better than the other with the data used in this analysis, therefore, we will only discuss ZINB models in this manuscript.

A PvDMFA-specific ZINB model (R^2^ = 0.956) was generated using results of PvDMFA where normal human AB + serum was tested (a total of 2,226 mosquitoes tested in 105 COMs by 63 independent assays), and the data allowed estimation of feed-to-feed and COM-to-COM variations (true %TRA of all normal serum COMs was considered to be zero). The details of PfSMFA data and analysis have been published before^17,20,23^. As expected from Fig. 1a,c, the negative binomial dispersion parameter θ are similar for PvDMFA and PfSMFA (Table 2, Model #1 vs. #4). On the other hand, zero-inflation parameter π, and the random effects of feed (Vf) and COM (Vc) are higher in PvDMFA compared to PfSMFA. For example, a model with π = 0.179 means that we expect that regardless of the amount of antibody present (or absent), at least 17.9% of the mosquitoes will not produce any oocysts. Higher Vf and Vc indicate that larger feed-to-feed and COM-to-COM variations, respectively, are predicted in PvDMFA than in PfSMFA when the same sample is tested in the same number of repeat feeds or multiple COMs (in a feed).

**Table 2:** Best fit parameters of ZINB models.

Model #	Assay	Exclude^a^	m_o_-contl^b^ (95% CI^g^)	π^c^ (95% CI^g^)	θ^d^ (95% CI^g^)	Vf^e^ (95% CI^g^)	Vc^f^ (95% CI^g^)
1	PvDMFA	No	9.6 (5.2–17.7)	0.179 (0.154–0.209)	1.698 (1.547–1.864)	5.822 (3.980–8.515)	0.326 (0.189–0.560)
2	PvDMFA	1	26.8 (17.4–41.3)	0.175 (0.150–0.205)	1.735 (1.582–1.904)	2.289 (1.502–3.488)	0.144 (0.077–0.269)
3	PvDMFA	4	37.6 (25.1–56.3)	0.158 (0.134–0.185)	1.793 (1.636–1.964)	1.773 (1.139–2.760)	0.081 (0.041–0.161)
4	PfSMFA	No	18.1 (16.2–20.2)	0.058 (0.053–0.063)	1.899 (1.845–1.955)	0.799 (0.666–0.957)	0.049 (0.039–0.061)
5	PfSMFA	1	18.6 (16.8–20.6)	0.058 (0.053–0.063)	1.909 (1.854–1.965)	0.676 (0.563–0.811)	0.041 (0.033–0.051)
6	PfSMFA	4	20.7 (19.0–22.7)	0.056 (0.051–0.061)	1.951 (1.896–2.009)	0.482 (0.399–0.582)	0.024 (0.018–0.032)

^a^Exclude feeding data from model building if mean control oocyst is lower than the specified value.

^b^Mean (arithmetic mean) oocyst in control.

^c^Zero-inflation parameter.

^d^Negative binomial dispersion parameter.

^e^Variance of the random effects for feed.

^f^Variance of the random effects for COM.

^g^95% confidence interval.

**Figure 2:**
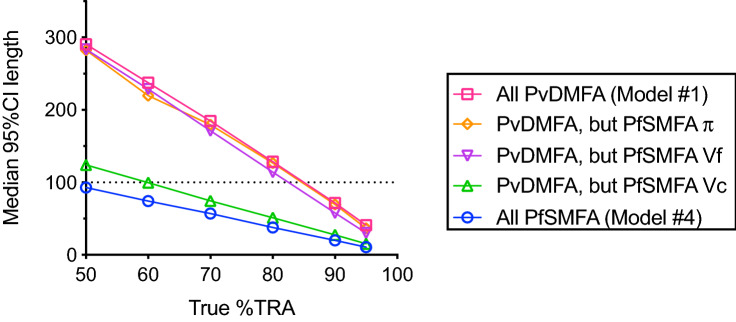
Impact of each parameter in ZINB model on accuracy of %TRA estimates. At each level of true %TRA (50, 60, 70, 80, 90 and 95% TRA), median of 95% confidence interval (95% CI) length of %TRA estimate was calculated from 10,000 iterations for each scenario of simulation, assuming 20 mosquitoes were analyzed in each COM (1 COM for control and 1 COM for test) in a single feeding assay. Simulations were performed using all 5 parameters (i.e., m_o_-contl, π, θ, Vf and Vc) from PvDMFA (red, Model #1 in Table 2) or from PfSMFA (blue, Model #4 in Table 2) models. Then each of π, Vf or Vc parameters in the PvDMFA was altered to the corresponding parameter in PfSMFA model (one parameter per time).

We next assessed the impact of each of the best-fit parameters in Model #1 (PvDMFA, red in Fig. 2) and #4 (PfSMFA, blue in Fig. 2) on %TRA estimations by simulations. In each iteration of the simulation, the best estimate %TRA and the 95% confidence interval (95% CI) for a test was calculated. From the 95% CI values, 95% CI length was determined as an indicator of the accuracy. For example, when a sample is tested by a feeding assay, and the high end of 95% CI (H95% CI) is estimated as 85% TRA, and the low end of 95% CI (L95% CI) as 60% TRA, the 95% CI length is calculated as 85–60 = 25. The median value of the 95% CI length (y-axis in Fig. 2) was obtained from 10,000 iterations for each scenario. PvDMFA data showed bigger 95% CI length than that in PfSMFA (Fig. 2), indicating more uncertainty in the %TRA estimates. In addition to the accuracy of %TRA estimate (how large or small was the 95% CI length), it is equally important to assess whether an observed inhibition is significant or not. Since the highest end of %TRA is 100% by definition (i.e., test COM has no oocysts), H95% CI cannot exceed 100% as well. Therefore, if 95% CI length of a test sample becomes larger than 100 (above the dotted horizontal line in Fig. 2), the L95% CI should be lower than 0% TRA, i.e., the observed %TRA is not significantly different from no inhibition. For example, the median 95% CI length of Model #1 at 80% TRA was 129. The result suggests that when a sample with true 80% TRA activity is tested in multiple PvDMFA assays (when the assays are performed as the simulated assay condition), at least in half of the assays, the observed inhibitions are judged as insignificant (i.e., L95% CI is lower than zero). The actual proportion of feeding experiments which show significant inhibitions is shown in Supplementary Fig. S1. Further simulations were performed to determine how the 3 parameters (π, Vf and Vc), which differed between PvDMFA and PfSMFA, effect the accuracy by switching each parameter of Model #1 to the corresponding one in Model #4. As clearly shown in Fig. 2, when the Vc parameter (COM-to-COM variation within a feed) from the PvDMFA model was replaced with that in PfSMFA model, the median 95% CI length reduced significantly, almost similar to the 95% CI length seen in the PfSMFA model (Model #4). On the other hand, replacing π or Vf values from PvDMFA model to those in PfSMFA model had minimum impact on the 95% CI length. The results clearly showed that higher Vc in PvDMFA model had the most influential effect on the uncertainty (i.e., bigger 95% CI length) in %TRA estimates, not higher π or Vf. In other words, if one modifies the assay, by which Vc can be reduced, the accuracy of %TRA estimates should be increased dramatically.

**Figure 3:**
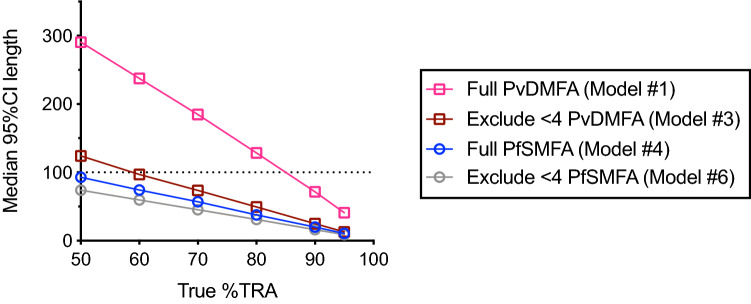
Impact of exclusion of lower m_o_-contl feeds on accuracy of %TRA estimates. As in Fig. 2, at each level of true %TRA, median 95%CI length of %TRA estimate was calculated from 10,000 iterations for each scenario of simulation, assuming 20 mosquitoes were analyzed in each COM (1 COM for control and 1 COM for test) in a single feeding assay. Simulations were performed using the 5 parameters (m_o_-contl, π, θ, Vf and Vc) from Model #1, 3, 4 and 6 in Table 2.

Previous studies with PfSMFA^16,20^ have shown that higher mean control oocysts (m_o_-contl) yields more accurate estimates of %TRA (if all the other conditions are the same). Therefore, modified models were generated by excluding feeding data with lower m_o_-contl (Model #2, 3, 5 and 6 in Table 2). In the analysis, two different cut-off values of exclusion were evaluated; 1 (i.e., excluded all COMs with < 1 m_o_-contl) and 4 (i.e., excluded COMs with < 4 m_o_-contl). In both PvDMFA and PfSMFA, Vf and Vc parameters went down gradually by increasing the cut-off value of exclusion from zero (no exclusion, Models #1 and 4) to 1 (Models #2 and 5) to 4 (Models # 3 and 6). The advantage of those modified models was assessed by additional simulations (Fig. 3). When feeding data with less than 4 m_o_-contl were excluded from the analysis (Model #3), the median 95% CI lengths became close to PfSMFA data. In the Model #3, the median 95%CI length line crossed y = 100 (black dotted line in Fig. 3) at ~ 60% TRA level of activity, instead of ~ 85% TRA in Model #1. The proportion of feeds which demonstrate significant inhibitions is shown in Supplementary Fig. S2.

When the cut-off value was increased more (e.g., 5 or 10, instead of 4), Vc (and also Vf and π) was further decreased, i.e., it increased the accuracy of %TRA estimates, as expected (data not shown). However, at this moment, there is no reliable way to keep m_o_-contl at a certain level (e.g., 4 or higher) in any given feeding experiment for DMFA and SMFA. Therefore, if the exclusion threshold is set to be higher, it reduces the throughput of assay (i.e., discard more DMFA or SMFA results when the control group does not reach to the determined threshold level of mean oocyst density). The best balance of throughput (reduce the threshold) and accuracy of %TRA estimates (increase the threshold) should be determined based on the usage of the assay. The results emphasize the importance of reporting %TRA estimates with the error ranges (e.g., 95% CI), not only presenting observed %TRA values, especially when the error range of %TRA estimates is large (e.g., assay with high COM-to-COM variation, using a lower exclusion threshold). In the remainder of this article, the threshold of 4 was selected, as we thought it was a practically acceptable criterion. In other words, Model #3 was used for all following simulations unless specified, and any real PvDMFA data which came from feeds with < 4 m_o_-contl were excluded from analysis.

### Two different readouts: TRA and TBA

**Figure 4:**
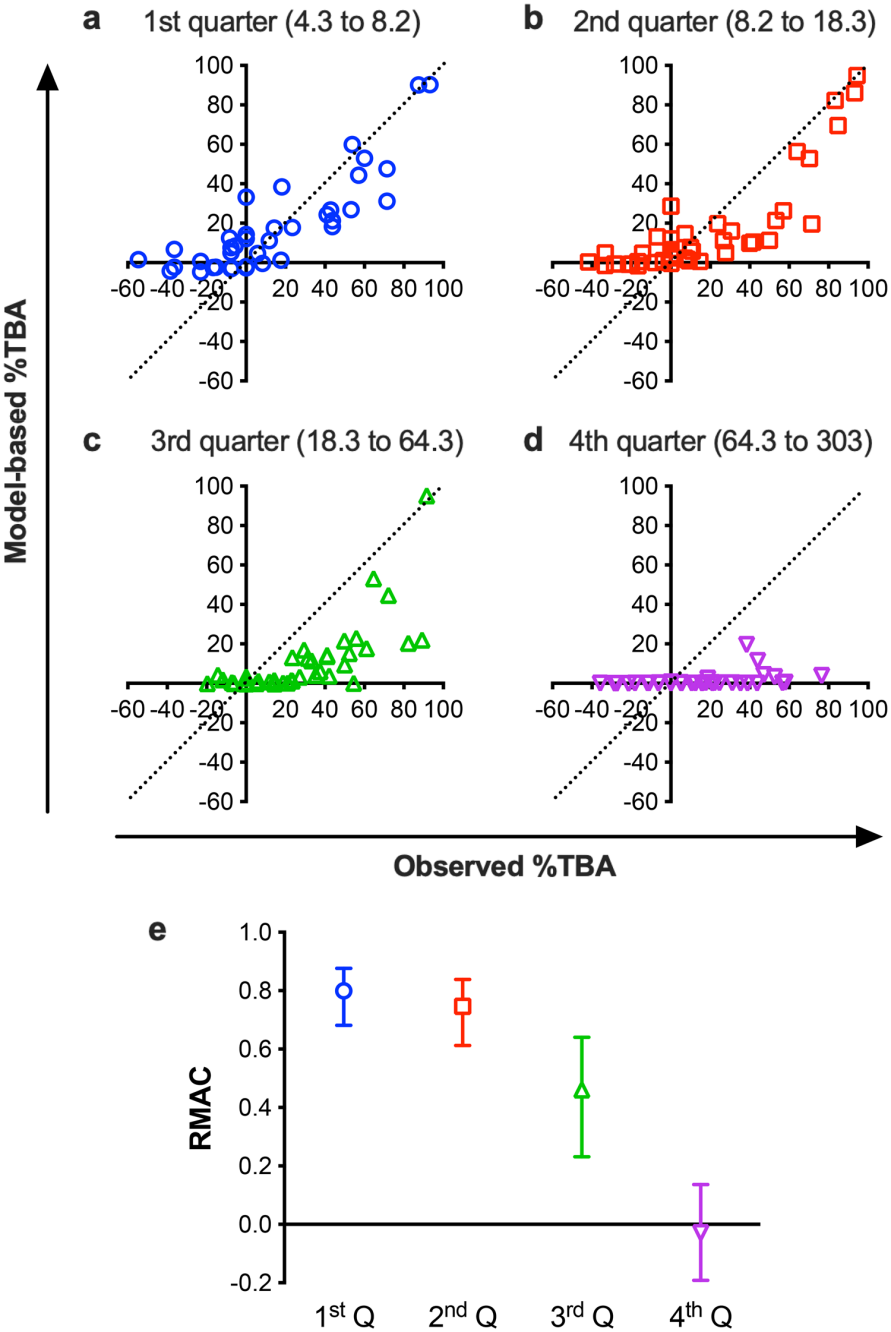
Concordance between observed %TBA and model-based %TBA. Using data from 161 COM pairs (a control and a test COM which were fed in the same assay) tested in 20 independent PvDMFA, model-based %TBA was calculated for each pair using observed %TRA and observed m_o_-contl values as published for PfSMFA^20^. The 161 data points were separated into 4 groups based on the m_o_-contl values: 1st, 2nd, 3rd and 4th quartile (Q), each group consists of 40 or 41 data points. The cut-off values of m_o_-contl were 8.2, 18.3 and 64.3 (minimum and maximum m_o_-contl for this analysis were 4.3 and 303, respectively). Figure 4a–d show individual COM pair data, and Fig. 4e shows concordance correlation (RMAC) of each group with the 95% CI.

Two different readouts have been used to express SMFA and DMFA results; %inhibition in oocyst intensity (%TRA) and %inhibition in prevalence of infected mosquitoes (%TBA). In the case of PfSMFA, it has been shown that %TBA results cannot be interpreted well without m_o_-contl data, while %TRA is independent of the readout from the m_o_-contl (at least at the level of m_o_-contl evaluated in the studies)^16,20^. More importantly, when model-based %TBA was calculated from observed %TRA and observed m_o_-contl using the ZINB model, there was a strong concordance between the observed %TBA and model-based %TBA^20^. The strong concordance has proved that %TBA and %TRA are not independent readouts, and %TBA may be determined by %TRA, m_o_-contl and the ZINB model. To assess whether there is the same interaction between %TRA, m_o_-contl and %TBA in PvDMFA, observed %TBA and model-based %TBA were compared (Fig. 4).

As shown in Fig. 4a (m_o_-contl ranged from 4.3 to 8.2) and Fig. 4b (m_o_-contl ranged from 8.2 to 18.3) when m_o_-contl were lower, there were strong concordances between observed %TBA and model-based %TBA as seen in PfSMFA^20^: RMAC = 0.80 and 0.75 for 1st quarter (Q) and 2nd Q, respectively (p < 0.0001 for both). The results indicate again that %TBA is a correlate of m_o_-contl and %TRA, and that %TBA and %TRA are not independent measurements, even for PvDMFA. However, when m_o_-contl became higher (3rd Q, m_o_-contl ranged from 18.3 to 64.3; Fig. 4c,e), the RMAC value went down (RMAC = 0.46, p < 0.001), and low and insignificant concordance was observed at the highest m_o_-contl group (4th Q, m_o_-contl ranged from 64.3 to 303; Fig. 4d,e, RMAC = − 0.03, p = 0.73). In theory, %TBA converges on zero when m_o_-contl becomes bigger when the same sample is tested. For example, if a control group has 300 oocysts on average, even if a test antibody reduces the average number of oocysts by 90% (i.e., 90% TRA), the average oocysts of the test group is 30, and the proportion of no-oocyst mosquitoes should be very minor (i.e., 0% TBA). As predicted, the majority of model-based %TBA values were ~ 0% in Fig. 4d, on the other hand, observed %TBA varied from − 36 to 77%. Why was there a discrepancy between observed %TRA and model-based %TBA values at higher m_o_-contl data set? As shown in Table 2, zero-inflation parameter π is bigger in PvDMFA than in PfSMFA. In other words, regardless of antibody activity (or no activity), more mosquitoes in a COM are likely to have zero oocysts in PvDMFA. Therefore, in PvDMFA, even when average oocyst number of a control COM is high, such as 4th Q in Fig. 4, a larger proportion of mosquitoes (both in control and test COMs) display zero oocysts by chance, not due to the transmission-blocking activity of test sample, compared to PfSMFA. While the effect of such zero-oocyst-mosquitoes on mean oocysts (which is used to calculate %TRA) is relatively smaller in a higher m_o_-contl feed, the zero-oocyst mosquitoes directly change observed %TBA values. Therefore, we hypothesized that higher π parameter in PvDMFA led to larger errors in observed %TBA values. To test the hypothesis, simulations were performed at various π levels.

**Figure 5:**
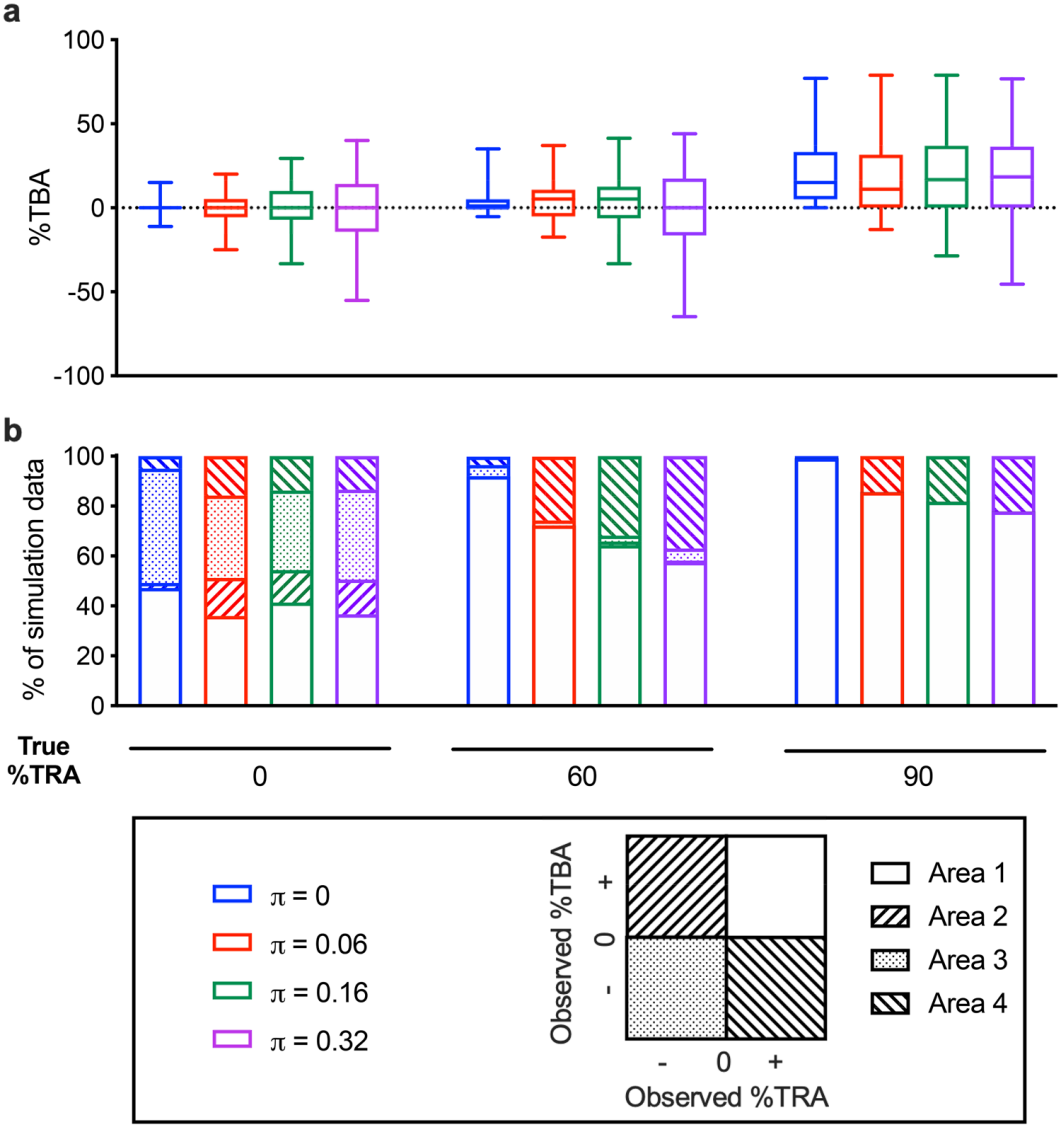
Impact of π parameter on observed %TBA. Assuming 20 mosquitoes are dissected per COM (1 COM for control and 1 COM for test) in a single feeding assay, simulations were performed with 4 different levels of π parameters (0, 0.6, 0.16 and 0.32) while keeping the other 4 parameters (i.e., m_o_-contl, θ, Vf and Vc) as Model #3. Test samples with 3 different %TRA levels (0, 60 and 90%TRA) were evaluated. (**a**) 25, 50, and 75 (bottom, middle, top of box) with 2.5 and 97.5 (lower and upper error bars) percentiles of %TBA from 500 iterations for each scenario are shown. (**b**) For each iteration, the simulation result was categorized into one of 4 groups based on the observed %TRA and observed %TBA: Area 1, %TRA ≥ 0 & %TBA ≥ 0; Area 2, %TRA < 0 & %TBA > 0; Area 3, %TRA < 0 & %TBA $\le$ 0; Area 4, %TRA > 0 & %TBA < 0. From 500 iterations in each scenario, percentage of results which were categorized into each area is shown.

Four different levels of π parameter (no zero-inflation, π = 0; same as PfSMFA, π = 0.06; same as PvDMFA, π = 0.16; and doubling for PvDMFA, π = 0.32) were tested with three different levels of %TRA samples (no inhibition, %TRA = 0; moderate inhibition, %TRA = 60, and strong inhibition, %TRA = 90). The other 4 parameters (i.e., m_o_-contl, θ, Vf and Vc) were kept as in Model #3. In all 3 levels of %TRA, %TBA values diverged more as π became bigger (Fig. 5a), indicating more uncertainty in %TBA estimates. Next, the proportion of the data set which were categorized into one of four areas was evaluated (Fig. 5b). Theoretically, all data should be categorized into “Area 1 (%TRA ≥ 0 & %TBA ≥ 0)” (when inhibitory samples are tested) or "Area 3 (%TRA < 0 & %TBA $\le$ 0)" (when enhancing samples are tested), but due to the error of assay, some data fell into "Area 2 (%TRA < 0 & %TBA > 0)" and "Area 4 (%TRA > 0 & %TBA < 0)". When 0% TRA sample was tested, %TRA and %TBA values could be positive or negative just by chance, and the simulation results were scattered into all 4 areas as predicted (but a majority of the data were within Areas 1 and 3). When an inhibitory sample was tested (%TRA = 60 or 90 in this case), in theory (i.e., if there is no error in the assay) all data should be classified into Area 1, and indeed, the largest proportion of data set in all scenarios was grouped into Area 1. However, a considerable number of data sets fell into Area 4 even with π = 0.06 (same level as PfSMFA), and the proportion further increased as π parameter was larger (Fig. 5b). When a DMFA (or SMFA) result happens to fall into Area 3 or 4 classes, an investigator might conclude the result as “enhancement” of infection (prevalence) when they use observed %TBA readouts without considering the error of the assay. Similarly, Area 2 or 3 data could be considered as “enhancement” of oocyst density. However, the simulation showed that the proportion of Area 2 or 3 results was much smaller than that in Area 4 when an inhibitory sample was tested (true %TRA of 60 or 90). These simulations strongly suggest that observed %TBA could be very misleading, especially for an assay with higher π.

**Figure 6:**
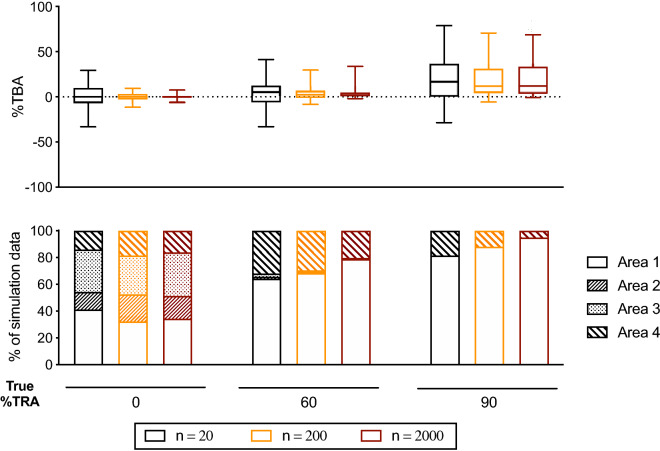
Impact of number of mosquitoes dissected on observed %TBA. Simulations were performed with 5 parameters (i.e., m_o_-contl, θ, π, Vf and Vc) from Model #3 as Fig. 5. Number of mosquitoes examined per COM (n) were 20 (same as Fig. 5), 200 and 2,000. The simulations assumed 1 COM for control and 1 COM for test were examined in a single feeding assay.

If the issue of using observed %TBA as a PvDMFA readout is the random error in measurement for prevalence of infected mosquitoes, one might think that the problem could be overcome by examining more mosquitoes per COM, not 20 mosquitoes per COM as in Fig. 5. Therefore, the impact of number of mosquitoes on %TBA readout was next assessed with the fixed π parameter (0.16). As expected, %TBA values diverged less when more mosquitoes were dissected (Fig. 6a). However, even when 2,000 mosquitoes per COM were examined, ~ 20% and ~ 5% of data fell into Area 4 at 60% or 90% TRA, respectively (Fig. 6b). Since dissecting 2,000 (or more) mosquitoes per COM is practically impossible, it is more realistic to use %TRA as the main readout of PvDMFA. Of note, as shown in previous studies with PfSMFA^16,20^, observed %TRA values are more reliable at higher m_o_-contl, but opposite for %TBA; observed %TBA is less reliable at higher m_o_-contl (Fig. 4).

### Impact of assay modifications on accuracy of %TRA estimates

Lastly, the impact of assay modifications on %TRA estimates was evaluated. At 4 levels of m_o_-contl (minimum number recommended, 4; 25th percentile of PvDMFA data which were used to generate Model #3, 10.4; 50th percentile, 22.1; and 75th percentile, 95.6), 3 levels of %TRA (60, 80 and 90%) samples were examined in 9 different assay conditions (number of mosquitoes dissected per COM was 20, 40 or 60; the same test sample was evaluated in 1, 2 or 3 independent feeding experiments).

**Figure 7:**
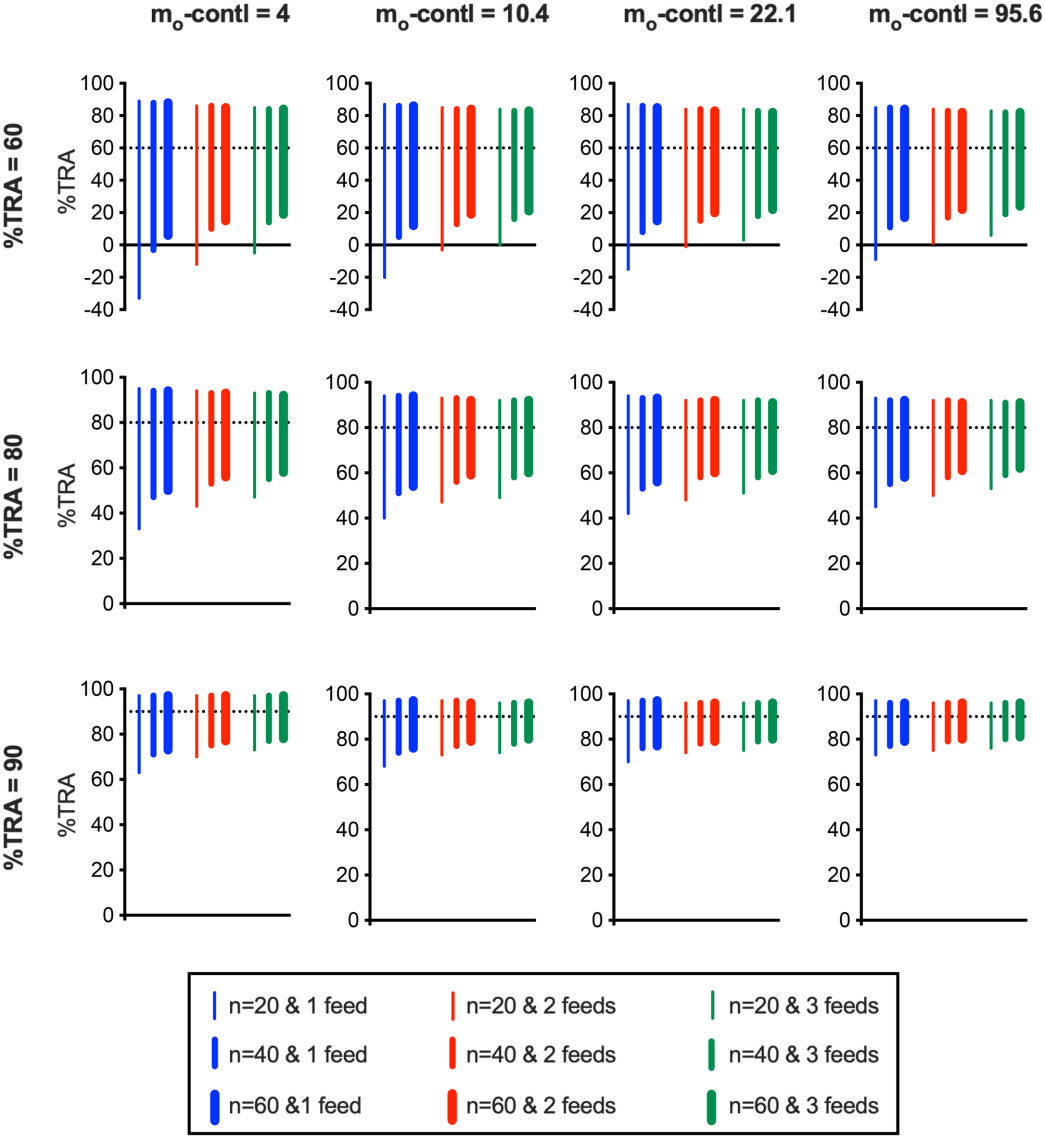
Impact of assay modification on %TRA estimates. Simulations were performed at 4 different levels of m_o_-contl (4, 10.4, 22.1 or 95.6) for 3 different levels of %TRA samples (60, 80 or 90) using the other 4 parameters (i.e., θ, π, Vf and Vc) from Model #3. A total of 9 different assay conditions were evaluated; number of mosquitoes examined per COM (n) was 20, 40 or 60 (1 COM for control and 1 COM for test), and each test sample was assessed by 1, 2 or 3 independent feeding experiments (feed). In each iteration, the low and high end of 95% CI of %TRA estimate (L95% CI and H95% CI, respectively) was calculated. From 10,000 iterations (per scenario), median L95% CI and H95% CI were calculated and the range between median L95% CI and median H95% CI is shown in the figure.

Figure 7 shows median of L95% CI and H95% CI of %TRA estimate from 10,000 iterations (per scenario). For example, when a test sample with 60% TRA activity was examined in a single feed and n = 20 per COM (20 mosquitoes were dissected in the control group, and another 20 mosquitoes for test group), and the control group in the feed showed 4 oocysts on average (m_o_-contl = 4), median L95% CI was calculated as − 33, and H95% CI was 89 (top left panel in Fig. 7). Since the L95% CI was lower than zero (no inhibition), it indicates that a sample with 60% TRA activity is unlikely to show a significant inhibition in at least > 50% of feeds. As predicted, more mosquitoes, more feeds, and higher m_o_-contl could increase the accuracy of the %TRA estimate (shorter bars in Fig. 7). When m_o_-contl is very low, such as < 4 oocysts on average, a small change in the m_o_-contl substantially affects the accuracy of %TRA estimate (Fig. 3 and previous studies^16,17^). However, the 95% CI lengths in m_o_-contl = 10.4 scenarios were not so different from those in m_o_-contl = 4 scenarios when the same sample was tested with the same test condition. Similar minor differences were observed for m_o_-contl = 10.4 vs. 22.1, or 22.1 vs. 95.6. The results suggest that it is not so critical to obtain higher m_o_-contl once it reaches a certain level. In these simulations, up to 60 mosquitoes per COM, and up to 3 repeat feeds were evaluated. For the laboratories which routinely perform PvDMFA, such modifications are considered as “doable” options, and some laboratories might do more. However, those require more time and resources, and a benefit of the modification (i.e., how much of 95% CI length can be narrowed down) is reduced as %TRA level goes up. For example, when a 60% TRA sample was tested at m_o_-contl = 4 (top left panel in Fig. 7), the 95% CI range was − 33 to 89% with n = 20 × 1 feed, and 19 to 84% with n = 60 × 3 feeds. By dissecting 9 times more mosquitoes, the median L95% CI became > 0 (i.e., now more than 50% of the time, one can call that the observed inhibition is significantly better than no inhibition), and the 95% CI length turned into 65 (= 84 − 19; n = 60 × 3 feeds) from 122 (= 98 − (− 33); n = 20 × 1 feed). On the other hand, with the same modification, the reduction in 95%CI length was only 15 (= 34 − 19) when a 90% TRA sample was tested (bottom left panel in Fig. 7). Depending on the target level of inhibitory activity which the investigators want to detect and desired accuracy in %TRA estimates, the methodology of PvDMFA (e.g., number of mosquitoes, repeat feeds) should be optimized, and simulations, such as shown in Fig. 7, will support the design of the assay. Not only for this purpose, the simulation results also help a proper interpretation of an observed %TRA value when the error range is not specified in the original report. As shown in Fig. 3, the median 95% CI length in PfSMFA and PvDMFA are reasonably close when assays with fewer mean control oocysts (i.e., < 4 oocysts on average in the simulations) are excluded. It suggests that investigators could use the results shown in Fig. 7 (or similar simulations) to estimate an error range of an observed %TRA either from SMFA or DMFA, or either with *P. falciparum* or *P. vivax*.

To the best of our knowledge, this is the first study which evaluates the model-fit and the impact of each parameter for DMFA data. Since DMFA uses patient blood as the source of gametocytes, and each blood sample contains different parasite mixtures, it is predicted that PvDMFA would show higher assay-to-assay variation (Vf). However, a mechanism of higher π and Vc in PvDMFA was not investigated in this study, because many factors could contribute to the differences in the parameters (e.g., *P. falciparum* vs. *P. vivax*, *An. stephensi* vs. *An. dirus*, SMFA vs. DMFA). To uncover the mechanism(s), a much larger set of data (e.g., PfSMFA performed with *An. dirus*, PvDMFA with *An. stephensi*) are likely to be required. As shown in Fig. 2, Vc parameter has a substantial effect on accuracy of %TRA estimates, therefore, it is ideal to determine at least the Vc parameter for each specific DMFA (or SMFA) condition in each laboratory. A second point which is not evaluated in this study is applicability of a ZINB model for other transmission-blocking interventions. All of the PfSMFA and PvDMFA data were collected during TBV development; i.e., to evaluate biological activity of vaccine induced antibodies. These strong associations between mean-SD and between mean-prevalence were similarly reported in studies which included data from transmission-blocking drugs^16^ and transmission-blocking antimicrobial peptides^24^. Therefore, we think it is possible to apply a similar ZINB model to evaluate other transmission-blocking interventions, other than TBVs (although each parameter needs to be reevaluated). However, if an intervention has a totally different mechanism of action (e.g., killing only oocyst-stages of parasites, instead of targeting gametocyte/gamete/zygote/ookinete parasites), a different mathematical model may fit better than a ZINB model.

This study also shows multiple commonalities between PvDMFA and PfSMFA, despite the fact that there are significantly differences in the assays in terms of parasite species (*P. vivax* and *P. falciparum*), source of gametocytes (patients’ blood and cultured gametocytes), mosquito species (*An. dirus* and *An. stephensi*), and geography (Thailand and United States). This study strongly suggests that the multiple commonalities found in this study can be applied for any other membrane-feeding assays, at least assays with human malaria parasites. Those include; (1) both data types can be explained by a zero-inflated negative binomial model, (2) the negative binomial dispersion parameter θ was similar, (3) excluding feeds with lower control oocysts (m_o_-contl) increases accuracy of %TRA estimates, and (4) %TBA is determined by %TRA and m_o_-contl, not an independent readout. In addition, when one wants to optimize or standardize DMFA or SMFA, not only control oocysts (m_o_-contl), but also COM-to-COM variation (Vc) need to be evaluated during the process. Based on the common ground between DMFA and SMFA, it is recommended; (1) to report %TRA with a proper error range (e.g., 95% CI), and (2) not to use observed %TBA as a main readout, because %TRA gives fairer comparisons of feeding results from different experiments and/or different investigators. Furthermore, a recent study by Churcher et al. has demonstrated that the intensity of a mosquito infection is critically important to the success of transmission^25^. The results suggest that %TRA readout could be superior than %TBA even from a biological point of view. Nevertheless, if for any reason it is preferred to use %TBA readout, we recommend using “standardized %TBA”^17^ rather than observed %TBA. Under the ZINB model %TBA is reasonably estimated from observed %TRA and m_o_-contl in PvDMFA (Fig. 4a,b in this paper) and in PfSMFA (as published before^20^). The “standardized %TBA” is a model-based %TBA calculated from observed %TRA and a giving m_o_-contl level, which an investigator can specify any value.

This study should support development of future transmission-blocking vaccines, and likely transmission-blocking drugs as well, to understand the pros and cons of DMFA and SMFA, to help better designing and reporting for future DMFA and SMFA experiments, and to aid rational comparisons of different candidates by properly interpreting the DMFA and SMFA results even when an error range of observed %TRA value is not described in the original report.

## Methods

### Direct membrane feeding assay with *P. vivax*

Patients visiting malaria clinics in north-west Thailand were microscopically examined for their malaria infections. When diagnosed with *P. vivax* infection and ≥ 15 years old, they were invited to donate their blood for PvDMFA. The protocol for blood collection from the patients was approved by Ministry of Public Health Ethical Committee, Bangkok, Thailand (protocol # WRAIR1308), and written informed consent was obtained from all volunteers. The details of PvDMFA methodology have been published elsewhere^26^. In brief less than two hours after collection, heparinized blood was aliquoted at 350 μL per tube before being centrifuged, and the plasma was removed. The packed erythrocytes were washed with incomplete RPMI medium to reduce the impact of patient plasmas on infectivity of gametocytes. Each tube with packed erythrocytes mixed with 180 μL of test antibodies (either sera or purified IgG samples) and a pool of normal human AB + serum was immediately placed in feeding apparatuses and offered to *Anopheles dirus* mosquitoes. The mosquitoes were allowed to feed on infected blood for 30 min. The blood samples were kept at 37–38 °C as much as possible using a temperature-control container during blood transportation and a waterbath during blood processing and feeding. After removal of unfed mosquitoes, remaining mosquitoes were kept at the insectary at 28 °C for 7 to 9 days before dissection. The oocysts in the midguts were examined and counted by microscopy. All methods were performed in accordance with the relevant guidelines and regulations. The original oocyst count in each individual mosquito is seen in Supplementary Table S1.

### Model building and statistical analysis

The ZINB model displayed in Table 2 was parameterized like the representative count model as described before^17^ by R with the package glmmTMB^27^. For Figs. 2, 3, and 7, data was simulated using the described parameters in different scenarios. In each iteration for each scenario of simulation, the 95% CIs were calculated using the ZINB-RE Sim 1 method as described in^17^. For Fig. 4, model-based %TBA was calculated as described in^17^ and the random marginal agreement coefficients (RMAC) analysis was performed^28^. RMAC is an agreement measure that is on the same scale as correlations (− 1 to 1), with 0 being agreement observed is the same as chance, and 1 being perfect agreement. The RMAC code is available upon request. The number of iterations in each scenario of simulation was described in the corresponding figure legend.

## Supplementary information

Supplementary Figures.

Supplementary Table S1.

## Data Availability

All original PvDMFA data are included in Supplementary Table S1.
