# Supplementary material for: Evaluation and modeling of direct membrane-feeding assay with Plasmodium vivax to support development of transmission blocking vaccines
Source: Sci Rep. 2020 Jul 28;10:12569. doi: 10.1038/s41598-020-69513-x (PMC7387523; doi:10.1038/s41598-020-69513-x)
Supplement: Supplementary file 1 — Supplementary Figures. [file 41598_2020_69513_MOESM1_ESM.docx]

**Supplemental material**

**Evaluation and modeling of direct membrane-feeding assay with *Plasmodium vivax* to support development of transmission blocking vaccines**

Kazutoyo Miura, Bruce J. Swihart, Michael P. Fay, Chalermpon Kumpitak, Kirakorn Kiattibutr, Jetsumon Sattabongkot, Carole A. Long

**Fig S1: Impact of each parameter in ZINB model on significance of %TRA estimates.** The simulations were performed as described in Fig. 2. Instead of median of 95% confidence interval (95%CI) length of %TRA estimate, the proportion of simulation results (from 10,000 iterations for each scenario) where the low end of 95%CI (L95%CI) was higher than zero (no inhibition) is shown in y-axis.

**Fig S2: Impact of exclusion of lower m_o_-contl** **feeds on significance of %TRA estimates.** The simulations were performed as described in Fig. 3. Instead of median of 95% confidence interval (95%CI) length of %TRA estimate, the proportion of simulation results (from 10,000 iterations for each scenario) where the low end of 95%CI (L95%CI) was higher than zero (no inhibition) is shown in y-axis.
